# Piloting an immersive virtual reality 3D self‐management education experience for adults with type 1 and type 2 diabetes mellitus: A qualitative study

**DOI:** 10.1111/dme.70383

**Published:** 2026-07-08

**Authors:** Marion A. Waite, Olga Kozlowska, Clare Martin, Michal Krezalek, David Duce, Arantza Aldea, Mark Green

**Affiliations:** ^1^ Oxford School of Nursing & Midwifery, Faculty of Health, Science & Technology Oxford Brookes University Oxford UK; ^2^ School of Engineering, Computing and Mathematics, Faculty of Health, Science & Technology Oxford Brookes University Oxford UK

**Keywords:** avatar, diabetes mellitus, self‐management education, technology, type 1, type 2, virtual reality

## Abstract

**Aims:**

Diabetes is a condition of global concern that can be mitigated through self‐management education. Virtual reality (VR) technology offers an immersive and interactive approach. It may promote self‐care and access to healthcare information. This study aimed to explore the experiences of adult participants with Type 1 or Type 2 diabetes during the piloting of a 3D Automatic Virtual Reality Environment (VR‐CAVE) approach to diabetes education. The study objective was to capture the participants' ideas for the future development of VR technology as a diabetes educational tool.

**Methods:**

We recruited fifteen adults with type 1 or 2 diabetes and invited them to a short diabetes education module in the VR‐CAVE. Subsequently, we undertook qualitative focus group interviews to elicit and evaluate the participants' experiences of various design elements and their considerations for future development.

**Results:**

Three categories emerged: ‘Experience of an immersive learning environment for diabetes education’, ‘Comparison with prior diabetes educational experiences’ and ‘Imagined development of the VR‐CAVE technology’. The participants reported a novel experience and a better understanding of the physiology of diabetes. The presence of an avatar felt non‐judgemental.

**Conclusion:**

Participants are open to using a VR‐CAVE for diabetes education. This study identifies aspects that could be enhanced through an immersive environment. Further research is needed to realise the benefits for the diabetes community and scale‐up the virtual reality resources and technology for different platforms to enable access for individuals and health service organisations.

**What's new?**

- Previous research shows that immersive self‐management education on smartphones as an intervention for people with diabetes (PWD) is acceptable and accessible.
- This study found that a 3‐D virtual reality (VR) environment for self‐management education offered PWD a novel learning experience about the physiology of their condition. Participants appreciated the instructor‐type avatar for being non‐judgemental and more engaging than traditional methods; this led them to identify self‐management topics suitable for simulation in VR.
- Additional research is necessary in partnership with PWD and service providers to establish suitable self‐management curricula and assess the scalability of VR resources for accessible platforms.

## 1 INTRODUCTION

There are 422 million people worldwide with diabetes, and the number has been sharply increasing for the past 30 years.^1^ Diabetes is a chronic condition with potential physical and mental health consequences. Kar argues,^2^ that technology can help people with diabetes (PWD) by providing preventive information and improving healthcare access, while enhancing the input of diabetes‐trained professionals. Ideally, the integration of technology into diabetes self‐management involves the diabetes community as a whole, including PWD, as well as their families, carers, healthcare professionals, including physicians, diabetes specialist nurses, dieticians, psychologists and diabetes educators.^3^

Virtual Reality (VR) is a computer‐generated simulated sensory experience where user actions can impact virtual surroundings.^4^ While there is incomplete evidence of practical learning gains,^4^ exploring the benefits for adult educational settings through the development of VR technology is possible. Some argue that VR has the potential to overcome language, literacy and numeracy barriers and lead to behavioural change,^5^ which is essential attributes of diabetes self‐management education. There are various reported examples of VR‐enhanced diabetes education, including improvements in cultural self‐efficacy,^6^ addressing diabetes and obesity,^7^ preventing type 2 diabetes (T2D), offering personalised training to improve physical activity,^7^, ^8^ improving cognitive function and plasma glucose levels,^7^ improving balance and reducing falls.^9^

More knowledge is needed to determine which groups or individuals benefit from VR and to identify unwanted effects. This paper reports a pilot study to explore the potential of a VR‐CAVE automatic virtual environment (VR‐CAVE) as a platform to deliver diabetes education to individuals with type 1 diabetes (T1D) and type 2 diabetes (T2D), and to identify requirements for further development based on users' experiences.

## 2 BACKGROUND AND CONTEXT

The current study is foregrounded by previous research undertaken by team members on an avatar‐based diabetes VR program.^10^ The study demonstrated the potential of immersive technology to deliver health education to PWD in Colombia, highlighting the importance of involving diabetes specialist clinicians such as endocrinologists in the content development process. We recognise that in other contexts, diabetes specialist clinicians include diabetes specialist nurses, dietitians, psychologists and diabetes educators.

Following the previous study, the investigating academic institution acquired a VR‐CAVE, which enabled the study team to create the study materials for a new educational space and explore how its features could enrich the experience. The study team comprised an interdisciplinary group of nursing, psychology and computer science professionals. The VR‐CAVE is a room where three‐dimensional (3D) images are projected onto three walls and viewed using 3D glasses.

Unlike the immersive headsets used in traditional VR, the VR‐CAVE glasses are transparent meaning that users can see their physical surroundings and interact with each other inside a 3D world. Such environments offer opportunities for collaborative learning.^11^ Appendix S1 details the VR‐CAVE system hardware, software and immersive learning design.

The current research adapted the diabetes resources (hypoglycaemia module) from the previous avatar‐based study with further input from National Health Service (NHS) diabetes specialist clinicians to add a carbohydrate sorting exercise inspired by UK structured education for PWD,^12^ with categories sourced from an NHS‐endorsed book.^13^ A quiz, based on educational information from Diabetes UK,^14^ was also incorporated. The resources were also influenced through consultation with a local diabetes patient and a public involvement and engagement group. Subsequent redesigns of the VR content aligned with theoretical recommendations for pedagogy when implementing immersive educational environments.^11^

The study aimed to explore the participants' experiences of the VR‐CAVE technology as an approach to diabetes education and capture their ideas for future development.

## 3 METHODOLOGY

The study team used a descriptive qualitative approach to gather early user feedback on VR‐CAVE development.^15^, ^16^ We were guided by the consolidated criteria for reporting qualitative research (COREQ)^17^ (Appendix S2) and qualitative research in healthcare informatics.^18^

Data collection methods focused on the participants' study visit to ‘the VR‐CAVE’, where they undertook one‐to‐one training sessions and the diabetes module.

### 3.1 Participant sampling

We used purposive sampling to recruit adults aged 16–80 with type 1 or type 2 diabetes who had been diagnosed with diabetes for at least 1 year. Exclusions included pregnancy, significant visual impairment or a co‐dependent relationship with a study team member. Participant study information was disseminated concurrently through social media and local community settings such as GP practices, pharmacies and libraries. Guided by recommendations for focus group qualitative research in human factors studies, we aimed to recruit 16–20 participants.^19^ Ethical approval was sought and obtained from the Oxford Brookes University research ethics committee (UREC Registration No: 231666).

Forty‐six people responded to the advertisement, and 16 participants were selected based on availability. Before the study visit, participants completed a pre‐study questionnaire on demographics and technology use. None of the participants had a pre‐existing relationship with any research team member. Researchers introduced themselves as an interdisciplinary team with a research programme on technology for diabetes care and self‐management.

### 3.2 VR‐CAVE training

After an initial welcome, group members were introduced to the VR‐CAVE (Figure 1).

**FIGURE 1:**
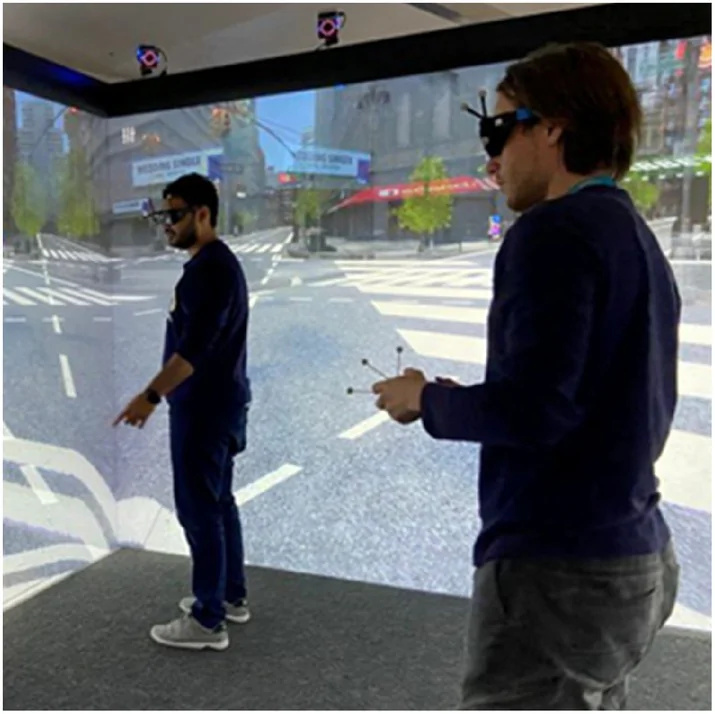
Users (not participants) interacting with the VR‐Cave.

The instructor demonstrated the tracked headset and controller. Participants did not wear headsets to access a clearer view of the 3‐D environment. Users were shown how to navigate, and a Quick Reference Guide (see Figure 2) was provided on the front wall of the VR‐CAVE. This aide‐memoire was created due to the participants' unfamiliarity with the VR‐CAVE controller's button configuration and effects. It was also intended to reduce cognitive load.

**FIGURE 2:**
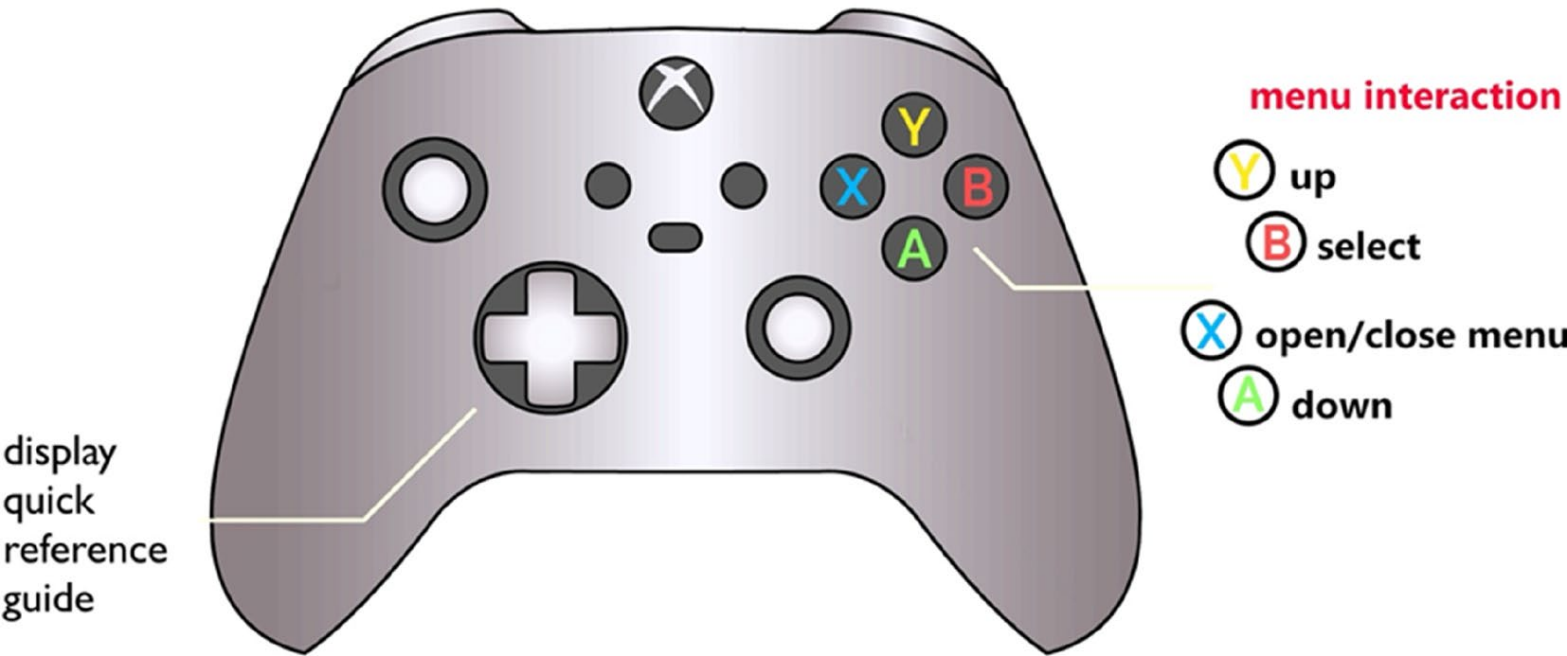
Quick reference guide.

### 3.3 Visit schedule

The participants were divided into four groups. Each group was designed for 3–5 participants, each attending only one visit. The investigators conducted the study visit according to a script adapted from Krug^20^ to ensure consistency of the user experience.

The study schedule was therefore repeated in an identical way four times. The itinerary is summarised in Figure 3, which also shows the activity duration and module topic (hypoglycaemia). Since the research objectives focused on user interaction and feedback, the User Evaluation component focused on simple interaction tasks, such as navigation and menu selection.

**FIGURE 3:**
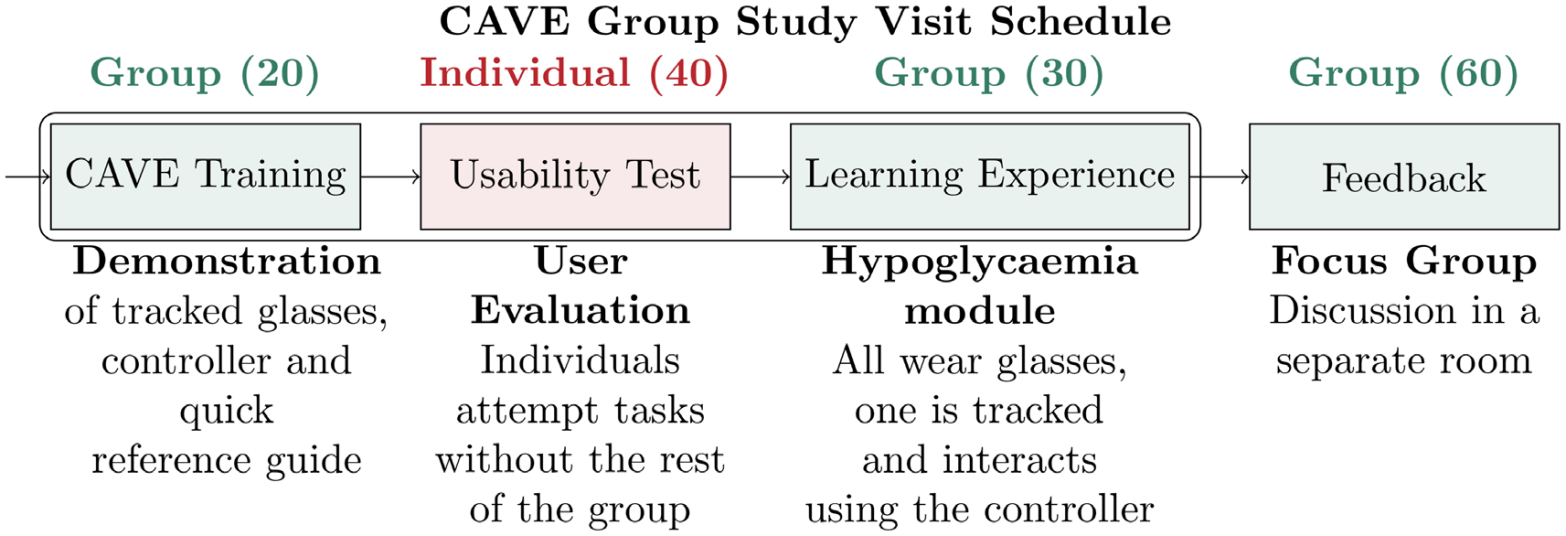
Participant visit schedule. Total activity duration (m) in parentheses.

### 3.4 Methods

After completing the diabetes module in the VR‐CAVE, the participants were invited to a qualitative focus group interview guided by a semi‐structured topic guide developed by the interdisciplinary study team. The interview questions focused on participants' experiences in the VR‐CAVE environment, their views on VR technology's affordances and challenges and a comparison of the VR‐CAVE with existing diabetes self‐management tools and desired improvements (Appendix S3). Two researchers (MW and OK) conducted the focus groups. The discussions started with outlining the ground rules to facilitate open discussion. The conversations lasting approximately one hour were recorded and transcribed verbatim. Transcripts were not returned to participants following the focus group. The researchers made field notes during the interviews.

### 3.5 Data analysis

Two research team members (MW and OK) independently coded the qualitative focus group data. The process commenced by reading the focus group interview transcripts and fieldnotes in full and noting initial thoughts about patterns in the data. The focus group transcripts were subsequently uploaded to NVivo (version 14.23.2) software for further analysis. The patterns and emergent codes were discussed multiple times to agree on the final categories.

The analytical process followed a qualitative descriptive design approach, focusing on straight descriptions of people's perspectives with minimal interpretation.^16^ Braun and Clarke's^21^ guidance on analysing qualitative data was drawn upon during the analysis.

Table 1 shows the code and category development.

**TABLE 1:** Category development.

Categories	Experience of an immersive learning environment for diabetes education	Comparison with prior diabetes educational experiences	Imagined development of the CAVE technology
Codes	Immersed and new way of learning	Receiving information	Physiological, physical, social and individualised factors
	Listening and human factor	Group working	Learning for various stages of the diabetes trajectory
	A visual way of learning	Less overwhelming than other diabetes programmes	Learning about diabetes with significant others
	High‐tech perception	Reflections on educational experiences with health service clinicians who are perceived as judgemental.	Cultural assumptions and valid resources
	Immersion in physiology promotes understanding.	The potential of an avatar as a peer with diabetes	Imagined possibilities of accessing 3‐D resources through different platforms
		Easier to assimilate information than website or paper‐based resources	Possibilities to appeal to different audiences, for example younger people

### 3.6 Researcher reflexivity

The researchers who undertook the focus group analysis are experienced early and mid‐career qualitative researchers in the use of healthcare technology for diabetes self‐management. We have previously worked in clinical roles specialising in diabetes, including psychology, nursing and education. To remain objective in our analysis of participants' reported experiences, we debriefed with each other and other study team members while acknowledging the potential impact of our own experiences.

## 4 FINDINGS

Fifteen PWD participated across four focus groups, one participant who had confirmed their attendance did not attend the scheduled study visit. Most participants were people with T1D (*n* = 10), with a mean diabetes duration of 13 years (5.5–21), and a mean of 7 (1.5–13) years since participating in a formal diabetes education programme. Participants' median age was 40 years, eight identified as men and seven as women.

All participant groups completed the diabetes module in the VR‐CAVE and solved most tasks despite having little experience with VR technology. The experience of diabetes education in the VR‐CAVE was reported as positive. While some shortcomings in content, delivery, usability, technology and environment were highlighted, overall, there was enthusiasm about the technology's potential. Three main and overlapping categories emerged from the data analysis and are reported here: ‘Experience of an immersive learning environment for diabetes education’, ‘Comparison with prior diabetes educational experiences’ and ‘The imagined development of the VR‐CAVE technology’.

### 4.1 Experience of an immersive learning environment for diabetes education

The ‘VR‐CAVE’ 3D environment was new to all participants, and full immersion in the content was perceived as a novel approach to diabetes education. Participants reported that visualising a 3D‐simulated blood vessel felt novel. It displayed glucose molecules circulating red blood cells and demonstrated the physiological process during hypoglycaemia:

Be inside the blood vessel. That was cool. It made it easy to understand. (G2, P2)

The experience was described as ‘high‐tech’, ‘visual’, ‘subconscious’, ‘safe’ and ‘engaging’:

You were getting drawn into a safe environment, a bubble, where it was visual… but at the same time, you're getting this other information coming through almost unconsciously, going in…. (G3, P3)

Participants described it as an immersive and new way of learning:

You will learn faster with visual feedback on how things work inside the body. With sugar inside, that's very easy to fix in your head. (G4, P2)

A further critical feature of the immersive environment reported by the participants was the presence of an avatar. Some participants described hearing an avatar that felt relatable and non‐clinical. The participants reported the VR‐CAVE avatar experience as straightforward, informative and non‐judgemental, with essential perceptions of not being talked at:

Not a nurse, not a doctor, but a voice talking to you about your diabetes. (G2, P1)

It's relatable because they're not an authority figure. (G2, P2)

Participants identified the potential accessibility of immersive approaches for diabetes education:

The real benefit is that it's on demand. You haven't got to wait three months for an appointment. (G1, P3)

### 4.2 Comparison with prior diabetes educational experiences

Participants compared their experiences of diabetes education in the VR‐CAVE with other resources for diabetes information. For some participants, the information felt easier to assimilate and less overwhelming:

I've picked up more today than remembering all the carb‐sorting and everything…. (G1, P3)

Individual preferences for receiving information were highlighted:

It depends on how you prefer to receive information or learn. I found that interactivity is motivational. Engaging, encouraging you to learn and explore. (G3, P4)

Comparative reflections included less favourable diabetes education approaches, such as those perceived as ‘dry’:

It was really interactive… better than just ticking boxes on a piece of paper. (G2, P2)

A leaflet that you might throw in a drawer. (G3, P4)

The perceived neutrality of the avatar prompted participants to report previous negative experiences of weight management education:

You get the lecture from the nurse, naughty, naughty you've gone over the borderlines, and you'll now have to take Metformin. And by the way, the threat of insulin is coming. You're almost wrist‐slapped, and you glaze over. (G1, P3)

Participants suggested that the VR‐CAVE could provide a non‐judgemental environment for this aspect of diabetes education. Structured group education is recommended for adults with diabetes in the UK NHS.^22^ Furthermore, some PWD belong to diabetes support groups or use social media to interact with other PWD. Participants compared their diabetes group experiences with collaborative learning in the VR‐CAVE, where they had to solve tasks as a group:

Before social media, you felt very much on your own, and that's lonely. So, coming to these sorts of things where you can engage with others sharing the same experience is really valuable. (G3, P3)

Some expressed gratitude for being a part of the group in solving the tasks and using the technology:

Personally, I'd rather be in a group because I like people… if you're in a group, then you can bounce off of each other. Something you say might trigger something in my mind and hopefully vice versa. So, I think groups generally are better. (G1, P1)

There was an acknowledgement that a group setting may not work for everyone, and it may not work in all situations:

It depends on what it is. If it was something that I'd never learnt about before, I would prefer to be on my own so that I could watch it back multiple times and not feel like I was annoying the other people who have got it on the first time. (G3, P3)

Some participants felt that the VR‐CAVE diabetes education approach did not meet their needs and preferred traditional methods:

I come from a much more word‐based and paper‐based generation. So, I learnt all about diabetes, in inverted commas, from a book or from a computer screen reading it. I'm not used to computer games. (G2, P5)

### 4.3 Imagined development of the VR‐CAVE technology

Participants' immersive learning experiences appeared to prompt imagined ideas for developing the VR‐CAVE for diabetes education, emphasising the physiological effects of diabetes and how physical and social actions and activities affect the person with diabetes. They suggested expanding the hypoglycaemia content to include how and why symptoms could be mitigated or corrected. including individualising scenarios, resources and content:

It could be easily visualised in VR‐CAVE what happens in the body when insulin is not present there when it should be there… what happens in the body of a person with and without diabetes… what is insulin, what is a pancreas. (G2, P1)

Why you feel hungry when you have low sugar and… how to mitigate it. (G2, P1)

The potential for visualisation prompted ideas about how the 3D aspects of the VR‐CAVE could be developed for individual use. Participants suggested more personalised content in the VR‐CAVE to understand the effects of different actions on their diabetes, such as what would happen if they did x, y or z. While reporting on the carb‐counting exercise or real‐life scenarios, the participants emphasised that their diabetes is ‘quite individual’ (G2, P5) and, therefore, requires a bespoke educational approach. A more individual and interactive approach was suggested with personalised examples:

You ate something, but you have taken too much insulin, and now you're doing a sport. What will happen? And then you can have different endings. (G2, P3)

It would be good to model that scenario by myself… I want to play with the figures, like, what would happen if I ate a biscuit? (G2, P1)

Integrating, for example, the Libre™… let's eat a bunch of cheese inside the VR‐CAVE and see what happens. (G4, P2)

Participants' suggestions extended beyond the topics covered in the diabetes module, highlighting the potential of VR‐CAVE technology for other areas of diabetes education. For example, to teach about *‘*different diabetes types’ (G4, P3). There was reported interest in material on how to manage one's diabetes on an everyday basis:

How to use the test machine… how to inject… how to manage your diet… exercise. (G1, P1)

The VR‐CAVE was perceived as enabling real‐life experiences:

It can teach you how to do things, rather than just telling you. (G4, P1)

Trying to calculate how much insulin you perhaps need to take for this meal, and the meal comes up on the screen, and you select. (G4, P3)

Familiarisation with the unknown elements of diabetes care was another suggestion:

People living with diabetes may benefit from developing scenarios of eye screening and annual checks, including foot checks so that people who “hate going to them” might be wondering what on earth it is because you don't know until you go. (G2, P2)

Participants recognised the potential value of VR‐CAVE education for learning at the various stages of a diabetes trajectory:

It looked very useful for a newly‐diagnosed diabetic in particular, even we learned some things, didn't we? (G3, P1 and P2)

Participants also suggested the VR‐CAVE technology and education would be suitable for younger people:

Young people want to have something interactive and playful to learn things. (G2, P3)

It would be a really cool way to learn. (G2, P2)

Group work in the VR‐CAVE appeared to trigger ideas about how immersive technology could be developed to include collaborative education with significant others:

You can have more than one person to do roleplay, so maybe one is having a hypo, so someone needs to go to the fridge and select the correct item to treat the hypo, a Coca‐Cola, but not cheese and not bread because it's slow. So you can go and pick the correct thing, so you are doing roleplay while he's having a hypo in the chair. (G4, P2)

Most participants described themselves as generally well‐informed about their diabetes and self‐management. There was some critique of the validity and cultural assumptions of the diabetes module content:

We disagreed with the results of one of the items, which was bread that looked like a white bread product, which is fast carbs, and it said that it was slow carbs, that's potentially a bit dangerous. (G3, P1)

A Coke can was labelled as soda, which is an American term, and so it could mean soda water or a high‐sugar Coke‐type drink. (G3, P3)

Some participants have suggested that reflecting the diversity of PWD is important for future development.

## 5 DISCUSSION

### 5.1 Summary of findings

The study aimed to explore the participants' experiences using the VR‐CAVE technology as an approach to diabetes education and capture their ideas for future development.

First, the study found that participants perceived a deeper understanding of their diabetes physiology through the immersive VR‐CAVE learning environment. While it may not suit everyone's preferences, participants viewed it as a secure and interactive way to simulate various diabetes‐related situations, including treatment, exercise, diet and collaborative learning with family and peers who do not have diabetes. In a previous study conducted by team members,^10^ hypoglycaemia was identified as a topic that needs more attention and understanding by people with PWD. In that study, participants reported improved self‐knowledge regarding how diabetes affects the body after interacting with VR technology. Our study provides additional evidence that immersive technology can help people better understand the impact of diabetes on the body and, potentially, the causes of various symptoms and how they can be managed. Scavarelli et al.^4^ argue that as technology improves and covers sensory modalities, the more immersive it becomes for the user and may improve a sense of embodiment, which may be critical in enhancing the agency of PWD.

Second, the study found that VR‐CAVE experiences encouraged participants to reflect on previous diabetes education, which many felt was judgemental and dry compared to the more engaging and peer‐like approach of the VR‐CAVE through the presence of an avatar. While this method may not suit everyone, it highlights important questions about who should contribute to delivering diabetes education and which strategies are most effective for various individuals and situations.

We have presented the results of our study to local clinicians, who identify the potential of the resources and technology for this group. They recognise that some aspects of structured diabetes education regarding physiology are dry and challenging to teach, and that the immersive approach might be more engaging.

Third, the study highlighted participants' ideas for the VR‐CAVE, focusing on various stages of diabetes and collaborative learning. Participants advocated for an immersive approach to diabetes education, particularly for younger individuals or those newly diagnosed. Structured education for all PWD in the United Kingdom post‐diagnosis is recommended.^23^

### 5.2 Comparison with existing VR studies in diabetes and long‐term conditions

Several studies have explored the use of VR interventions in the context of diabetes and other chronic conditions, highlighting both the therapeutic potential and limitations of such approaches. For instance, Lee & Shin,^24^ demonstrated that a VR‐based exercise programme significantly improved balance and reduced postural sway in elderly individuals with type 2 diabetes, suggesting its utility in fall prevention. Similarly, Yim & Hur^25^ found that while VR interventions did not lead to reductions in HbA1c, they enhanced balance and self‐efficacy in fall prevention, reinforcing the motivational and engagement benefits of VR for diabetes self care.

Expanding beyond diabetes‐specific outcomes, Skip Rizzo et al.^6^ discussed the broader application of VR and exergaming technologies in addressing obesity and promoting physical activity. Although current exergames may not meet recommended exercise intensity levels, they offer a promising adjunct to traditional exercise, particularly for populations at risk of metabolic conditions.

Neira‐Tovar et al.^26^ contributed a design framework for VR games tailored to healthcare, emphasising the importance of integrating motivational elements to enhance patient engagement. Their study showed that VR tools could support physical health and reduce stress in participants with chronic diseases, including type 2 diabetes, through well‐designed, user‐centred virtual environments.

Van Der Kruk et al.^27^ in their scoping review of VR as a patient education tool in long‐term health conditions suggest the potential benefits of VR in assisting patient education but argue that there remains a need to identify which groups or individuals benefit from VR. However, healthcare professionals should consider using VR as an adjunct to patient education.

Together, these studies underscore the growing evidence base for VR as a supportive tool in managing diabetes and other long‐term conditions. They emphasise the importance of engagement and motivational design in VR interventions, aligning with the current study's aims to explore the future development of VR technology as a diabetes educational tool.

### 5.3 Implications for educating diabetes healthcare professionals

While this project does have the potential to support medical students and healthcare professionals in understanding the physiological aspects of diabetes, a more significant insight emerged from our participants' narratives: they frequently highlighted the attitudes of healthcare professionals towards diabetes education as a barrier to effective care. This underscores the importance of addressing not only clinical knowledge but also the psychosocial dimensions of diabetes management.

Recent studies have shown that VR can be a powerful educational tool for healthcare professionals, particularly in fostering empathy and awareness of the social determinants of health. For example, Beverly et al.^28^ utilised cinematic VR to immerse diabetes care providers in patient experiences, thereby improving their cultural self‐efficacy and understanding of patient priorities. Reynolds et al.^29^ found that VR modules enhanced empathy and positively shifted attitudes among physician assistant students towards participants with diabetes. Persky et al.^30^ further demonstrated how VR simulations can reveal and potentially mitigate biases in clinical encounters, especially those related to race and obesity.

These findings support the idea that immersive VR experiences can help healthcare professionals better appreciate the lived experiences of PWD, challenge negative attitudes and promote more person‐centred care—aligning closely with the concerns voiced by participants in our study.

### 5.4 Future work

Type 2 diabetes in young adults (18–40 years) is rapidly increasing in the United Kingdom, in alignment with the increased global prevalence and incidence of diabetes.^31^ These individuals do not fit neatly into established diabetes services, which are either aimed at younger people with T1D or older people with T2D. Younger people with T2D face unique difficulties, such as a long duration of living with diabetes, increased vascular risk compared to their type 1 peers and often the additional challenges of living with obesity and deprivation. Education close to diagnosis aims to support lifestyle change for diabetes management. Offering structured education at diagnosis is a quality standard in the NICE guidelines, as measured in the annual National Diabetes Audit.^31^ However, despite this, the uptake of diabetes‐structured education in the first 1–2 years after diagnosis remains extremely low.^31^ Younger people with T2D could benefit from exploring immersive approaches like the VR‐CAVE. This could help engage them with the learning design and take advantage of the technology's potential for simulation.

As the study demonstrated, the participants recognised the collaborative potential of the VR‐CAVE technology for simulation and how it could be applied to scenarios where it would be beneficial to learn alongside family members or peers who do not have diabetes. The participants' suggestions are worthy of more exploration, and further patient and public engagement with the technology could inform future studies and development.

The participants appeared to assume that the VR‐CAVE afforded access to information related to diabetes self‐care that could be used individually. Future work will explore how resources could be transferred between platforms that are easily accessible and scalable for different contexts, especially healthcare providers.

While participants noted some cultural shortcomings in the current VR‐CAVE diabetes resources, it is arguably possible to design simulated, culturally appropriate contexts by working with wider groups.

### 5.5 Limitations

The study was small‐scale, and participants were recruited from the general adult diabetes population. Further studies are needed to determine which demographic groups of PWD might benefit most. A previous study investigated the feasibility of avatar‐based VR technology for educating individuals with T2D.^9^ However, it occurred in a different geographical and socio‐economic context, with technological variations and was designed for individual learning. The findings of both studies suggest the possibility of access and engagement with diabetes education that may be available to complement extant healthcare provider approaches. There is potential to investigate further the educational differences between both approaches, including an economic cost‐benefit analysis of developing immersive technology for diabetes education. Critically, future research must evaluate how VR resources can be scaled up for use by healthcare providers, such as by creating transferrable resources across mobile VR platforms. Following the study, we have consulted with and presented the findings to PWD, diabetes charities, NHS diabetes care commissioners, other researchers in the field and industry experts. A shortcoming of the 3‐D VR Cave is its limited scalability due to the high cost of the hardware. However, a suggestion emerging from the public engagement work is to utilise the VR‐Cave to develop, pilot and test diabetes education resources that may be transferable to scalable and lower‐cost platforms, such as those used in our previous study.

As the study showed, immersive technology is not for everyone; therefore, it is essential to consider approaches involving wider groups of PWD to determine which educational delivery methods are acceptable to different individuals and circumstances.

## 6 CONCLUSION

Technology is vital in promoting self‐care, health behaviours and well‐being for PWD.^22^ Our study demonstrates the potential capability of VR‐CAVE immersive technology to enhance diabetes education based on the users' suggestions of aspects of diabetes education and support that could be improved through an immersive environment. Simulated and collaborative learning could simulate different scenarios related to diabetes treatment, exercise, social situations and diet, providing a secure and interactive learning experience. However, the resources are at an early stage of development, and more knowledge is needed about their effectiveness and potential for wider‐scale use and dissemination.

## FUNDING INFORMATION

This work was supported by an Oxford Brookes University Research Excellence Award 22/23.

## CONFLICT OF INTEREST STATEMENT

The authors declare that they have no known competing financial interests or personal relationships that could have appeared to influence the work reported in this paper.

## Supporting information


Appendix S1.
