# Supplementary material for: Piloting an immersive virtual reality 3D self‐management education experience for adults with type 1 and type 2 diabetes mellitus: A qualitative study
Source: Diabet Med. 2026 Jul 8;43(11):e70383. doi: 10.1111/dme.70383 (PMC13669991; doi:10.1111/dme.70383)
Supplement: Supplementary file 1 [file DME-43-e70383-s001.rtf]

Appendix S1: further details on 'the CAVE' system hardware and software.


CAVE systems vary in complexity from 6-sided environments (walls, floor and ceiling) to 3-sided environments (3 walls). The CAVE used in this study was a 3-sided environment, with stereo images back-projected onto three large glass screens by three high-performance projectors, one per screen (see Fig 1.). The screen dimensions are 3.5m x 2.185m. A high-performance workstation drives each projector, and a seventh connects to the outside world. Users view the screens through 3D glasses. A unique feature of this set-up (though not used in this study) is that a camera system can track the positions and orientations of the glasses of two users, and hence, two users can see views projected from their viewpoints. One user uses a game controller to interact with the environment.

Below is a diagrammatic representation of the CAVE showing the locations of the screens, projectors, and six tracking cameras.


Figure 1CAVE 3-D Environment
The educational content for the study was presented using the widely available Unity 3D engine. Unity 3D was connected to the CAVE using the open source UniCAVE package (1) adapted for this specific hardware configuration. Existing and new educational content was developed as a Unity application


References
1.	Tredinnick, R., B. Boettcher, S. Smith, S. Solovy, and K. Ponto, Uni-CAVE: A unity3d plugin for non-head mounted vr display systems, in 2017 IEEE Virtual Reality (VR), pp. 393–394, IEEE, 2017.


Appendix S2: Consolidated criteria for reporting qualitative studies (COREQ): 32-item checklist

Developed from:
Tong A, Sainsbury P, Craig J. Consolidated criteria for reporting qualitative research (COREQ): a 32-item checklist for interviews and focus groups. International Journal for Quality in Health Care. 2007. Volume 19, Number 6: pp. 349 – 357


No.  Item 
	Guide questions/description	Reported on Page #	
Domain 1: Research team and reﬂexivity 			
Personal Characteristics 			
1. Interviewer/facilitator	Which author/s conducted the interview or focus group? 	Page 6

	
2. Credentials	What were the researcher's credentials? E.g. PhD, MD 	Page 8

	
3. Occupation	What was their occupation at the time of the study? 	Page 8	
4. Gender	Was the researcher male or female? 	N/S
	
5. Experience and training	What experience or training did the researcher have? 	Page 8	
Relationship with participants 			
6. Relationship established	Was a relationship established prior to study commencement? 	Page 6
.  	
7. Participant knowledge of the interviewer 	What did the participants know about the researcher? e.g. personal goals, reasons for doing the research 	Page 5
	
8. Interviewer characteristics	What characteristics were reported about the inter viewer/facilitator? e.g. Bias, assumptions, reasons and interests in the research topic 	Page 8	


Domain 2: study design 		
	
Theoretical framework 		
	
9. Methodological orientation and Theory 	What methodological orientation was stated to underpin the study? e.g. grounded theory, discourse analysis, ethnography, phenomenology, content analysis 	Page 5	
Participant selection 		
	
10. Sampling	How were participants selected? e.g. purposive, convenience, consecutive, snowball 	Page 5
	
11. Method of approach	How were participants approached? e.g. face-to-face, telephone, mail, email 	Page 5

	
12. Sample size	How many participants were in the study? 	Page 6
	
13. Non-participation	How many people refused to participate or dropped out? Reasons? 	Page 6

	
Setting		
	
14. Setting of data collection	Where was the data collected? e.g. home, clinic, workplace 	Page 5 and page 6
.	
15. Presence of non-participants	Was anyone else present besides the participants and researchers? 	No	
16. Description of sample	What are the important characteristics of the sample? e.g. demographic data, date 	Page 6
	
Data collection 		
	
17. Interview guide	Were questions, prompts, guides provided by the authors? Was it pilot tested? 	Appendix 3

	
18. Repeat interviews	Were repeat interviews carried out? If yes, how many? 	Yes, four see page 6
	
19. Audio/visual recording	Did the research use audio or visual recording to collect the data? 	Page 7	
20. Field notes	Were ﬁeld notes made during and/or after the inter view or focus group?	Page 7	
21. Duration	What was the duration of the inter views or focus group? 	Page 7

	
22. Data saturation	Was data saturation discussed? 	No
	
23. Transcripts returned	Were transcripts returned to participants for comment and/or correction? 	No
 	
Domain 3: analysis and ﬁndings 			
Data analysis 	
		
24. Number of data coders	How many data coders coded the data? 	Page 7
	
25. Description of the coding tree	Did authors provide a description of the coding tree? 	Page 7 - table
	
26. Derivation of themes	Were themes identiﬁed in advance or derived from the data? 
	Page 7
	
27. Software	What software, if applicable, was used to manage the data? 	Page 7
	
28. Participant checking	Did participants provide feedback on the ﬁndings? 	No
	
Reporting 	
		
29. Quotations presented	Were participant quotations presented to illustrate the themes/ﬁndings? Was each quotation identiﬁed? e.g. participant number 
	Page 9 to 14

	
30. Data and ﬁndings consistent	Was there consistency between the data presented and the ﬁndings? 	 Yes, there was.
Page 9 to 14
	
31. Clarity of major themes	Were major themes clearly presented in the ﬁndings? 	Yes. they were.
From page 9 to 14
	
32. Clarity of minor themes	Is there a description of diverse cases or discussion of minor themes?      	Discussion of major and minor themes
From page 9 to 14	


Appendix S3: The focus group schedule.

Focus Group Agenda

INTRODUCTION 5 min	

1.	Greet/thank participants, housekeeping (eg phones on mute)
2.	Recap the purpose of the pilot program, noting that this only focuses on a specific subject within the full hypoglycaemia module
3.	Focus group ground rules 
a.	respect others' opinions
b.	listen without interrupting
c.	don't talk over each other
d.	maintain confidentiality
4.	Open the floor to discussion, guided by the following topics:

DISCUSSION 45 mins	

1: OVERALL FEEDBACK 

Tell us about your experience with the CAVE           

How did you interact with the system? 
How did you feel about the CAVE as a physical environment?     
How did you feel about being asked to complete the tasks in the CAVE? 

2: PILOT TOPIC OF CHOICE

Tell us about your previous experiences learning about/exploring hypoglycaemia, such as reading about it or as part of diabetes education. 

How do you feel about technology for diabetes care? 

Was it worthwhile for you to explore hypoglycaemia in the CAVE? 

How easy was it to understand what hypoglycaemia is? 
How easy was it to undertake the carb sorting activity? 

Do you feel that you have learnt something new about hypoglycaemia, how it affects you and how to manage it? 
Would it be useful to others?

3: Experiences, expectations and barriers 

Is there anything that could improve your experience? (enhancements) 
●	What enhancements to the system would you like to see to make it more useful to you? [Possible prompts: Different information, more interaction, home viewing via a headset].
●	Are there any enhancements that would make it more useful to others?
●	Is there anything else you would like to discuss that you haven't discussed?

What did you like about it? Did not like about it?

Are you concerned about using technology such as the CAVE in diabetes education?

Would you use it again, for example, for another topic? 
Would you see it as a valuable tool in diabetes education? Any ideas for other topics? 

Thinking about your previous experiences with 'diabetes education' - is there anything that could have been done better if CAVE technology had been used? 

How did you find being part of the group in the CAVE? Should it be more or less interactive with other participants? 

4: LANGUAGE AND APPROACH (the voice) 
Ask participants:
●	if they were satisfied with the tone, language, approach, level of maturity, etc
●	if they would change anything in particular

CLOSING COMMENTS 	
1.	Is there anything else you would like to add? 
2.	Thank participants for their time
